# A Phosphatidylserine Source of Docosahexanoic Acid Improves Neurodevelopment and Survival of Preterm Pigs

**DOI:** 10.3390/nu10050637

**Published:** 2018-05-18

**Authors:** Randal K. Buddington, Victor V. Chizhikov, Igor Y. Iskusnykh, Helen J. Sable, Jeffrey J. Sable, Zade R. Holloway, Tamar Blumenfeld Katzir, Marie van der Merwe, Taisiya Yakimkova, Karyl K. Buddington, Yael Lifshitz, Shoshi Tessler, Ariel Gilbert

**Affiliations:** 1 School of Health Studies, University of Memphis, Memphis, TN 38152, USA; mvndrmrw@memphis.edu (M.v.d.M.); tykmkova@memphis.edu (T.Y.); 2 Department of Anatomy and Neurobiology, College of Medicine, University of Tennessee Health Sciences Center, Memphis, TN 38163, USA; vchizhik@uthsc.edu (V.V.C.); iiskusny@uthsc.edu (I.Y.I.); 3 Department of Psychology, University of Memphis, Memphis, TN 38152, USA; hjsable@memphis.edu (H.J.S.); zrhllway@memphis.edu (Z.R.H.); 4 Department of Behavioral Sciences, Christian Brothers University, Memphis, TN 38104, USA; jsable@cbu.edu; 5 Department of Neurobiology, Tel Aviv University, Tel Aviv 6997801, Israel; tamarbkatzir@gmail.com; 6 Department of Biological Sciences, University of Memphis, Memphis, TN 38152, USA; kbudding@memphis.edu; 7 Enzymotec, Sagi 2000 Industrial Park, Migdal HaEmeq 23106, Israel; yael.lif.medved@gmail.com (Y.L.); shoshi.tessler@gmail.com (S.T.); gilbertariel@gmail.com (A.G.)

**Keywords:** prematurity, preterm infant, docosahexanoic acid, phosphatidylserine, brain, neurodevelopment

## Abstract

The amount, composition, and sources of nutrition support provided to preterm infants is critical for normal growth and development, and particularly for structural and functional neurodevelopment. Although omega-3 long chain polyunsaturated fatty acids (LC-PUFA), and particularly docosahexanoic acid (DHA), are considered of particular importance, results from clinical trials with preterm infants have been inconclusive because of ethical limitations and confounding variables. A translational large animal model is needed to understand the structural and functional responses to DHA. Neurodevelopment of preterm pigs was evaluated in response to feeding formulas to term-equivalent age supplemented with DHA attached to phosphatidylserine (PS-DHA) or sunflower oil as the placebo. Newborn term pigs were used as a control for normal in utero neurodevelopment. Supplementing formula with PS-DHA increased weight of the brain, and particularly the cerebellum, at term-equivalent age compared with placebo preterm pigs (*P*’s < 0.10 and 0.05 respectively), with a higher degree of myelination in all regions of the brain examined (all *p* < 0.06). Brains of pigs provided PS-DHA were similar in weight to newborn term pigs. Event-related brain potentials and performance in a novel object recognition test indicated the PS-DHA supplement accelerated development of sensory pathways and recognition memory compared with placebo preterm pigs. The PS-DHA did not increase weight gain, but was associated with higher survival. The benefits of PS-DHA include improving neurodevelopment and possibly improvement of survival, and justify further studies to define dose-response relations, compare benefits associated with other sources of DHA, and understand the mechanisms underlying the benefits and influences on the development of other tissues and organ systems.

## 1. Introduction

Normal growth and development of the preterm infant is dependent on adequate nutrition support. Yet, the specific needs and sources of nutrition remain uncertain and many preterm infants suffer ex utero growth retardation that compromises structural and functional development [1]. All organs are at risk with the brain considered particularly sensitive to inadequacies of the amount and composition of nutrition [2] as brain development in the third trimester and early neonatal period is rapid and dramatic [3].

Omega-3 long chain polyunsaturated fatty acids (LC-PUFA), and particularly docosahexanoic acid (DHA), have been linked to brain and central nervous system functions [4,5]. Overall, the majority of fetal DHA accretion is acquired in the third trimester, coinciding with brain and retina maturation [6,7]. While the potential benefits of providing supplemental DHA have been considered inconclusive for term [8] and preterm [9,10] infants, recent studies reported DHA is particularly critical for the premature infant [7], corroborating previous recommendations [11]. The lack of consistency in findings has been attributed to differences in when and how assessments are performed, the dose and source of supplementation, fatty acid composition of the test formulas, and the gestational age, health status, and postnatal environment of the preterm infants.

The source of LC-PUFA has been related to bioavailability. Phosphatidylserine (PS) is a naturally occurring phospholipid [12,13]. The greatest concentration of PS in humans is in the brain, where it comprises about 15% of the total phospholipid pool, with a structural role in maintaining the integrity of cell membranes [12]. PS is also considered critical for the functioning of neuron membranes, including signal transduction, secretory vesicle release, cell-to-cell communication, and cell growth regulation [13]. Compared with DHA associated with triacylglycerols, chronic administration of DHA esterified to PS (PS-DHA) results in greater DHA accretion in the cortices of middle-age rats [14], indicating improved DHA bioavailability. Importantly, human milk throughout lactation includes DHA conjugated to PS [15,16], suggestive of the importance of PS as a delivery mechanism.

To avoid the confounding factors and ethical limitations of using preterm infants as test subjects, we used preterm pigs as a relevant and translational preterm animal model for a comprehensive screening approach to evaluate if growth, survival, and neurologic structural and functional development to term equivalent age respond to supplementing formula with PS-DHA. Litters of preterm pigs allowed us to compare responses of siblings that share genetics, gestational age and uterine environment, and postnatal care, thereby reducing the confounding variables that compromise interpretations of clinical studies. The present findings will guide the design of additional studies to establish dose-response relationships for specific targeted outcomes.

## 2. Methods

### 2.1. Collection of Preterm and Term Pigs

All phases of the research using animals were approved by the University of Memphis Institutional Animal Care and Use Committee (approval date: 31 July 2015, approval number: 0748). Preterm pigs were harvested as before [17] by sterile caesarian section at 92% of gestation (gestation day 105 of 115 day term) from five specific pathogen-free sows of a consistent genetic lineage that were bred by artificial insemination. At this stage of gestation, the preterm pigs are considered relevant to 32-week preterm infants. An additional five pigs were collected immediately after natural delivery at term from two sows to obtain structural and functional data and serve as references for normal, full term development.

Within 3 to 4 h after preterm delivery, an umbilical artery catheter was placed (3.5 Fr, Umbili-Cath; Utah Medical Products, Midvale, UT, USA), a feeding tube (8 Fr; Kendall feeding tube and urethral catheter, Covidien, Mansfield, MA, USA) was inserted via the cheek, and both were secured. Three Ag/AgCl electrodes were secured to the head of each pig at this time using plastic cups that were modified to allow administration of electrode gel (SE-20, J & J Engineering, Poulsbo, WA, USA). (1) The positive electrode was placed on the vertex of the head (homologous to Cz in the international 10–20 system; [18]); (2) The negative (reference) electrode was placed posterior to the auditory meatus; (3) The ground electrode was placed anterior to the positive electrode on the forehead. As before [17], an all-in-one parenteral nutrition (PN) solution was provided beginning immediately after the umbilical artery catheter was placed. The pigs also received 5–6 mL of sow plasma to provide maternal antibodies and compensate for the lack of colostrum and no intrauterine transfer of passive immunity. The pigs were provided a prophylactic antibiotic (Cefazolin; 10 units/kg; IV; 2 times during the first 48 h). The PN solution was provided for only 16–18 h until the morning after delivery, and thereafter, the source of nutrition was entirely milk replacer.

### 2.2. Diets and Feeding

A commercial milk replacer (Soweena Litter Life; Merrick’s, Middleton, WI, USA) was prepared following the manufacturer’s instructions and fed via the feeding tube at a rate of 20 mL/kg every 3 h for a total volume of 160 mL/kg-day. The milk replacer fed to the PS-DHA pigs (*n* = 31) was supplemented with PS-DHA using InCog^®^ (PS-DHA group, InCog^®^, Enzymotec Ltd., Migdal HaEmek 23106, Israel, 190 mg/100 mL). At the volume fed this provided 58 mg of phosphotidylserine per kg-day. The milk replacer fed to the placebo pigs (*n* = 35) was supplemented with an equivalent amount of sunflower oil (190 mg/100 mL).

### 2.3. Growth, Health Status, and Survival

Each pig was observed at least every 3 h when fed and comments about activity, responsiveness, stool consistency, temperature, and general health were recorded. Body weights were recorded daily to adjust the volume of formula to be fed for the next 24 h. Pigs that became lethargic, non-responsive, with labored (agonal) breathing and poor circulation based on mottled skin were humanely euthanized and necropsied to determine the cause of morbidity. The remaining pigs were euthanized (Euthasol, 1 mL/4.5 kg, via the umbilical catheter) at term-equivalent age (10 days after delivery) and after nine days of enteral nutrition. Prior to death blood samples were collected for routine hematology, a serum metabolic panel that included lipids, and samples from the placebo and PS-DHA groups from two litters were used to measure cytokines at the time of collection and after 24 h exposure to lipopolysaccharide (MagPlex, ThermoFisher, Waltham, MA, USA).

### 2.4. Brain and Cerebellum Weight

The entire brain was removed and the weight was recorded before the cerebellum was isolated and weighed separately.

### 2.5. Magnetic Resonance Imaging of the Brain

Intact cerebral hemispheres of 3 placebo and 4 PS-DHA pigs that had been fixed in formalin were imaged using a 7T/30 Bruker Biospec scanner (Bruker, Billerica, MA, USA). Quantitative T2-3D images had a matric size of 128 × 68 and 66 slices were performed. Diffusion tensor imaging (DTI) included 50 slices in 60 directions. The resulting T2 and DTI indices were calculated using MATLAB (version 7.12, Mathworks Inc., Natick, MA, USA). MRI parameters were calculated using in-house software written in Matlab (©MathWorks, Natick, MA, USA).

### 2.6. Novel Object Recognition (NOR)

The NOR task is a test of cognition that evaluates recognition memory [19] and relies on an animal’s innate exploratory behavior [20]. NOR has previously been used successfully in pigs [21]. The NOR task was conducted eight days after delivery (PD 9; one day before necropsy) using a rectangular-shaped plexiglass chamber with mounts on each end to hold objects 5 cm off the floor within the visual range of the pigs. During the habituation phase a pig was placed in the center of the chamber for 20 min with identical objects secured to the mounts. The pig was then removed and returned to its incubator for 10 min. During this time, the chamber and all objects were thoroughly cleaned with diluted bleach and dried to remove any olfactory cues. One of the objects in the chamber was replaced by a novel object, with the location and type of novel object counterbalanced between the groups. The pig was then returned to the chamber for the 10-min test phase that was videotaped to determine the amount of time spent in physical contact with the familiar (old) and the novel (new) object. If a pig spent less than 60 s (1/6th of the test period) with either object, the data were considered to be an unreliable assessment of recognition memory and the pig was excluded. Novelty preference was determined by dividing the time in contact with the novel object during the test phase by the total time for the test phase, and converting the resulting quotient to a percentage. A higher score represents greater preference for the novel object and is indicative of greater recognition memory for the familiar object previously presented during the habituation phase [22].

### 2.7. Event-Related Brain Potentials

Preterm birth can adversely impact sensory processing and neural development, and is associated with an increased risk of hearing loss [23]. This led us to examine development of auditory event-related brain potentials (ERPs) to determine if supplementing with phosphatidylserine would alter the trajectory of development for the processing of auditory signals. ERPs were recorded immediately after delivery and on postnatal days 2, 5, and 10 (the morning of scheduled necropsies) in response to pairs of 50-ms tones (500-ms inter-tone interval) that were presented during a 30-min testing period with randomized inter-pair intervals (IPI) of 1 or 5 s. These measurements were made using 19 preterm (8 placebo and 11 PS-DHA) and 13 newborn term pigs. Processing, averaging, and analysis of the ERP (Biopac BSL, Biopac Systems, Goleta, CA, USA, and custom MATLAB scripts (MATLAB, version 7.12, Mathworks Inc., Natick, MA, USA) involved applying a bandpass filter of 0.1–30 Hz and segmentation of data into 1200-ms epochs, including 200-ms prior to onset of the first tone in each pair. Epochs with voltage > ±50 μV or no change in 10 data points were excluded from further analysis. The included epochs were averaged by interval and pig and each epoch was corrected relative to its prestimulus mean. Peak amplitudes and latencies were identified for each component in each pig; N1: most negative peak 50–150 ms after tone-pair onset and P2: most positive peak 150–250 ms after tone-pair onset. The data were analyzed using a mixed-design analysis of variance, with Greenhouse-Geisser corrections applied where appropriate. 

## 3. Results

### 3.1. Survival

The c-sections provided a total of 77 preterm pigs with 65 surviving the initial 24 h of parenteral nutrition (PN). One litter with 23 fetuses suffered five deaths shortly after delivery before the start of PN. Of the 65 pigs that survived the 24 h of PN, 43 survived to term equivalent age (66%). None of the pigs that died during the period of enteral nutrition exhibited signs of necrotizing enterocolitis and the cause(s) of death were not otherwise evident or determined. At term equivalent age survival for the placebo and PS-DHA pigs that started EN was 60 ± 15% and 75 ± 10%, respectively (*p* = 0.044). The mortality rate of preterm pigs exceeds that for preterm infants born at similar stages of development, reflecting the challenges associated with providing intensive care to entire litters. 

### 3.2. Growth

Body weight at the start of EN was similar for placebo and PS-DHA pigs (Table 1). Growth in body weight expressed as the ratio of final weight of individual pigs divided by the weight at the start of 9 days of EN tended to be higher for placebo compared to PS-DHA pigs (1.39 ± 0.04 vs. 1.29 ± 0.05; *p* = 0.097), but this did not result in a difference in final weight between the groups for pigs that completed the trial. Both groups of preterm pigs tended to be smaller at term equivalent age compared with newborn term pigs (*p* < 0.10).

### 3.3. Brain and Cerebellum Weights

The cerebella of preterm pigs fed the milk replacer supplemented with PS-DHA were heavier (*p* < 0.05) than those of placebo pigs (Table 1). There was a trend for the total brain weight to be greater for PS-DHA pigs (*p* = 0.08). Compared with newborn term pigs, total brain and cerebellum weights were similar for PS-DHA pigs, but were lower for placebo pigs. 

### 3.4. Blood Metabolic and Hematology Panels

The metabolic panels and hematology using blood collected immediately before necropsy were similar for PS-DHA and placebo pigs with the exceptions of higher sodium and tendency for lower aspartate aminotransferase for PS-DHA pigs (Tables S1 and S2). All values were within normal ranges we have previously measured in other studies using preterm pigs delivered at the same stage of gestation and raised to term equivalent age. Similarly, cytokine concentrations at the time of blood collection and the responses to lipopolysaccharide exposure did not differ between groups (data not presented).

### 3.5. Magnetic Resonance Imaging of the Brain

The T2 imaging of the brains revealed significant differences for diffusion tensor imaging indicies (*p* < 0.05) and tendencies to be different (*p* < 0.10) between groups with greater myelination in multiple regions of the PS-DHA pigs (Table 2). Brains of newborn term pigs were not imaged.

### 3.6. Novel Object Recognition

The criterion of a pig spending more than 60 s at one or both objects to be included in the analysis affected both groups equally, with final samples of eight placebo (57%) and nine PS-DHA (60%) pigs. During the recognition memory test, both groups had an overall preference for the novel object, with a higher percentage of time at the novel object for the PS-DHA pigs (75% ± 7 vs. 52% ± 10; *p* = 0.04).

### 3.7. Event-Related Brain Potentials

The sensory pathways and central processing of auditory inputs developed after delivery. In general, N1s became larger and were earlier with development (*P’s* = 0.04 and 0.03 for the main effect of post-delivery age on peak amplitude and peak latency, respectively). Peak amplitudes were highest on Day 5, but were more mature on Day 10. Across days, the N1 peak amplitude for the second tone was larger for placebo compared with PS-DHA pigs (*p* = 0.04), suggesting the supplement of PS-DHA enhanced inhibition of the second tone.

N1 peak amplitudes were similar for newborn full-term pigs (*p* = 0.463). However, the waveforms and the N1 peaks were more mature morphologically in the term-equivalent preterm pigs than in the newborn full-term pigs and peaked significantly earlier (*p* = 0.05).

The P2 peak amplitude increased with development and was larger after first tone than the second for the placebo pigs, but this pattern was not the case for the PSDHA group (*p* = 0.014 for comparison of placebo and PS-DHA pigs).

## 4. Discussion

This study using a clinically relevant large animal model demonstrated significant improvements in structural and functional indicators of neurodevelopment among preterm pigs fed formula supplemented with PS-DHA. This was evident by the increased cerebellum weight, enhanced myelination of white matter and the fiber organization, improved sensory processing, and cognition. Previous studies using preterm infants to demonstrate and understand the potential benefits of DHA and other nutrients for structural and functional neurodevelopment have been limited by ethical considerations and complicated by confounders. This may explain why supplementing the prenatal diet of women during pregnancy with DHA has not provided conclusive evidence for improving functional indicators of neurodevelopment of their infants [24]. Similarly, increasing the total fatty acids in breast milk or formula represented by DHA from 0.3% to 1% was not associated with a significant increase in intelligence quotient [10]. The present study avoided many of the confounders by using a clinically relevant large animal model and comparing siblings that shared genetics, gestational age, and in utero and ex utero environment. The findings demonstrate significant improvements in structural and functional indicators of neurodevelopment among preterm pigs fed formula supplemented with PS-DHA.

The weight of the fetal pig brain increases from 17.5 g to 25.5 g between gestational days 102 to 110 [25] and at term equivalent age (115 days post conception) was 31.4 g (present study). This rapid rate of growth later in gestation is similar to the growth of the human brain during the last trimester. An important consideration is how growth of the brain is spared at the expense of other tissues [26]. As a result, at a specific stage of development brain mass is relatively constant over a range of fetal and neonatal body weights. Therefore, the differences at term equivalent age between the placebo compared with PS-DHA and neonatal term pigs, but not between PS-DHA and newborn term pigs are significant and indicate the supplemental PS-DHA allowed for normal growth of the brain and particularly the cerebellum after preterm delivery. The imaging of the brains and improved myelination corroborated the improved structural neurodevelopment associated with the PS-DHA supplement. However, the slightly larger brains of pigs naturally delivered at term compared with PS-DHA pigs emphasize, the challenges associated with prematurity that can diminish brain growth, regardless of improved nutrition support. 

The neurologic immaturity and corresponding underdeveloped motor skills of preterm pigs poses challenges in assessing functional indicators of neurodevelopment that rely on movement. Despite the relatively small sample sizes after excluding pigs that did not remain at either object long enough to be included in the analysis, the PS-DHA pigs showed a greater interest in the novel object, suggesting greater development of recognition memory. Our assessment of ERP development is innovative for preterm pigs and yielded novel data that have not been previously reported. Importantly, the waveforms for the preterm pigs exhibited maturational development during the 10 day period and the emerging pattern with a relatively large N1 peak amplitude after a longer delay is similar to that for humans and other species [27]. The enhanced inhibition of the second tone detected with the PS-DHA pigs is indicative of effective attentional processes. Interestingly, the more mature ERP waveforms for the term equivalent preterm compared with the newborn full term pigs suggests preterm birth and early exposure to ex utero life may accelerate development of sensory pathways. The long term consequences are uncertain.

## 5. Conclusions

Collectively, the several data sets for structural and functional neurodevelopment of the preterm pig validate the preterm pig as a translational model for neurodevelopment and demonstrate a benefit of supplementing formula with PS-DHA. Additional studies are needed to define the dose-response relationship, evaluate if there are long-term benefits, and determine if PS-DHA provides additional benefits compared with other sources of DHA. Evidence is accumulating that supplementing the diet of preterm infants with DHA provides benefits that extend beyond neurodevelopment [28,29]. Corresponding with this, the higher survival of PS-DHA pigs in four of the five litters resulted in higher overall survival for as yet unknown reasons. The lack of differences for blood chemistries indicate little if any influence of PS-DHA on commonly used biomarkers for health and metabolic status, with a similar lack of response for hematology. Although supplementation of the maternal diet with DHA to increase fetal levels yields an anti-inflammatory response in term infants [30] and may provide similar benefits to preterm infants subjected to inflammatory challenges such as necrotizing enterocolitis and bronchopulmonary dysplasia [31], this benefit was not evident for the PS-DHA pigs based on the similar initial levels of cytokines and the responses to lipopolysaccharide.

## Figures and Tables

**Table 1:** Body weights of preterm and term pigs at the time of delivery and for preterm pigs at term equivalent age after nine days of enteral nutrition using milk replacer without (placebo) or with docosahexanoic acid (DHA) conjugated with phosphatidylserine (PS-DHA) and corresponding total brain and cerebellum weights at term equivalent age. Values are means and standard errors. Values in rows with different letter superscripts are different at *p* < 0.05. * is for the birth weight of pigs born at term.

Weight (g)	Placebo	PS-DHA	Term
Body weight at gestation day105	961 ± 42	983 ± 40	
Body weight at term equivalent age	1304 ± 49	1345 ± 57	1450 ± 60 *
Brain	30.0 ± 0.5 ^a^	31.4 ± 0.5 ^ab^	32.8 ± 0.4 ^b^
Cerebellum	4.37 ± 0.12 ^a^	4.85 ± 0.10 ^b^	5.07 ± 0.42 ^b^

**Table 2:** Diffusion tensor imaging indices for different regions of the brains of pigs fed milk replacer with docosahexanoic acid (DHA) conjugated with phosphatidylserine (PS-DHA) or placebo (means and standard deviations) with differences determined by *t*-test. The higher values of PS-DHA pigs are indicative of greater extent of myelination.

Region	PS-DHA	Placebo	*p* Value
Left Frontal Cortex	52.23 ± 1.31	42.89 ± 0.94	0.054
Right Frontal Cortex	51.67 ± 1.22	37.69 ± 0.76	0.011
Left Hippocampus	59.50 ± 2.93	39.74 ± 2.53	0.016
Right Hippocampus	55.91 ± 2.81	38.37 ± 2.22	0.008
Globus Pallidus	46.44 ± 0.35	34.13 ± 0.26	0.001
Hypothalamus	66.27 ± 3.26	52.09 ± 0.43	0.013
Thalamus	47.24 ± 0.58	36.25 ± 0.07	0.004
Corpus Callosum	49.34 ± 0.79	31.23 ± 0.27	0.059
Left Internal Capsule	43.77 ± 0.64	30.60 ± 0.38	0.052
Right Internal Capsule	42.70 ± 0.34	30.46 ± 0.29	0.032

## Supplementary Materials

The following are available online at http://www.mdpi.com/2072-6643/10/5/637/s1, Table S1: Serum chemistry values for blood collected from preterm pigs at term equivalent age after nine days of feeding the two milk replacers with docosahexanoic acid (DHA)-enriched phosphatidylserine (PS-DHA) or placebo, Table S2: Hematology of blood collected immediately before euthanasia from preterm pigs at term equivalent age after nine days of feeding the two milk replacers with docosahexanoic acid (DHA)-enriched phosphatidylserine (PS-DHA) or placebo.
