# Supplementary material for: A Phosphatidylserine Source of Docosahexanoic Acid Improves Neurodevelopment and Survival of Preterm Pigs
Source: Nutrients. 2018 May 18;10(5):637. doi: 10.3390/nu10050637 (PMC5986516; doi:10.3390/nu10050637)
Supplement: Supplementary file 1 [file nutrients-10-00637-s001.zip › nutrients-292672-SI.pdf]

Table S1. Serum chemistry values for blood collected from preterm pigs at term equivalent age after nine days of feeding the two milk replacers with docosahexanoic acid (DHA)-enriched phosphatidylserine (PS-DHA) or placebo.

| Parameter | Control | PS-DHA | *P* value |
|---|---|---|---|
| Glucose (mg/dL) | 67 ± 7 | 66 ± 10 | 0.99 |
| Blood Urea Nitrogen (mg/dL) | 15.1 ± 1.9 | 15.7 ± 2.5 | 0.84 |
| Creatinine (mg/dL) | 0.73 ± 0.04 | 0.72 ± 0.08 | 0.90 |
| Na (mmol/L) | 141 ± 0.6 | 145 ± 1.8 | 0.03 |
| K (mmol/L) | 4.7 ± 0.11 | 4.7 ± 0.23 | 0.97 |
| Cl (mmol/L) | 104 ± 1.2 | 106 ± 2.0 | 0.33 |
| Ca (mg/dL) | 9.3 ± 0.2 | 9.5 ± 0.2 | 0.35 |
| Protein (g/dL) | 3.3 ± 0.14 | 3.2 ± 0.14 | 0.57 |
| Albumin (g/dL) | 1.61 ± 0.20 | 1.53 ± 0.158 | 0.78 |
| Globulin (g/dL) | 1.47 ± 0.09 | 1.60 ± 0.10 | 0.34 |
| Bilirubin (mg/dL) | <0.20 | <0.20 | 1 |
| Alkaline Phosphatase (IU/L) | 678 ± 54 | 650 ± 59 | 0.77 |
| Aspartate Aminotransferase (IU/L) | 20.9 ± 2.2 | 16.7 ± 0.5 | 0.09 |
| Alanine Aminotransferase (IU/L) | 11.3 ± 1.4 | 12.5 ± 1.1 | 0.52 |
| Cholesterol (mg/dL) | 88.8 ± 4.8 | 92.7 ± 8.0 | 0.66 |
| Triglycerides (mg/dL) | 25.1 ± 2.0 | 25.0 ± 3.0 | 0.97 |
| HDL Cholesterol (mg/dL) | 53.8 ± 4.1 | 55.3 ± 5.8 | 0.83 |
| VLDL Cholesterol (mg/dL) | 6.3 ± 1.3 | 6.1 ± 1.0 | 0.90 |
| LDL Cholesterol (mg/dL) | 30.0 ± 2.3 | 31.5 ± 3.5 | 0.69 |

Table S2. Hematology of blood collected immediately before euthanasia from preterm pigs at term equivalent age after nine days of feeding the two milk replacers with docosahexanoic acid (DHA)-enriched phosphatidylserine (PS-DHA) or placebo.

| Parameter | Control | PS-DHA | *P* value |
|---|---|---|---|
| WBC $10^9$/L | 13.75 ± 2.52 | 11.38 ± 2.42 | 0.58 |
| Lymphocytes $10^9$/l (%) | 13.5 ± 2.6 (98 ± 1) | 11.2 ± 2.5 (98 ± 1) | 0.59 (0.81) |
| Monocytes $10^9$/L (%) | 0.07 ± 0.01 (0.6 ± 0.1) | 0.06 ± 0.01 (0.6 ± 0.1) | 0.65 (0.77) |
| Granulocytes $10^9$/L (%) | 0.05 ± 0.02 (0.6 ± 0.2) | 0.12 ± 0.03 (1.3 ± 0.6) | 0.22 (0.42) |
| Red Blood Cells $10^{12}$/L | 4.11 ± 0.38 | 4.86 ± 0.49 | 0.35 |
| Hematocrit | 22.6 ± 2.6 | 25.6 ± 2.4 | 0.49 |
| Hemoglobin g/dL | 7.04 ± 0.82 | 8.69 ± 1.01 | 0.32 |
| Platelets (10^9/l) | 274 ± 63 | 240 ± 31 | 0.66 |
